# Dynamics Analysis of a New Fractional-Order Hopfield Neural Network with Delay and Its Generalized Projective Synchronization

**DOI:** 10.3390/e21010001

**Published:** 2018-12-20

**Authors:** Han-Ping Hu, Jia-Kun Wang, Fei-Long Xie

**Affiliations:** 1 School of Automation, Huazhong University of Science and Technology, 1037 Luoyu Road, Wuhan 430074, China; 2 State Key Laboratory of Cryptology, P.O. Box 5159, Beijing 100878, China; 3 Key Laboratory of Image Information Processing and Intelligent Control, Ministry of Education, Wuhan 430074, China

**Keywords:** dynamics analysis, fractional-order, Hopfield neural network, generalized projective synchronization

## Abstract

In this paper, a new three-dimensional fractional-order Hopfield-type neural network with delay is proposed. The system has a unique equilibrium point at the origin, which is a saddle point with index two, hence unstable. Intermittent chaos is found in this system. The complex dynamics are analyzed both theoretically and numerically, including intermittent chaos, periodicity, and stability. Those phenomena are confirmed by phase portraits, bifurcation diagrams, and the Largest Lyapunov exponent. Furthermore, a synchronization method based on the state observer is proposed to synchronize a class of time-delayed fractional-order Hopfield-type neural networks.

## 1. Introduction

Fractional calculus has a history of 300 years. In the early days, because of its computational complexity and lack of intuitive physical and geometric explanations, it did not attract the interest of researchers [1,2,3]. Recently, people have discovered that using fractional calculus to describe many natural phenomena will be more accurate, such as biomedical engineering [4], fractional control [5], and specific physical problems [6]. The fractional calculus model is considered as an excellent tool to describe the hereditary and memory characteristics of various processes due to a memory term in the model [1].

The Hopfield neural network (HNN), which can store memories like the human brain does, is considered as one of the most influential neural networks [7]. It is natural to consider that incorporating the two memory terms (fractional calculus and the Hopfield neural network) will potentially be a huge step toward the enhancement of both memory characteristics and the efficiency of information processing [8]. Studies have shown that chaos, which appears in the human brain, plays a vital role in memory storage in a biological neural network [9,10,11]. It is known to us that there exists a delay effect on information processing in intra-neural communication. Therefore, studying the chaotic fractional-order Hopfield neural network (FHNN) with time-delay is a very significant issue that can narrow the gap between biological neuronal systems and artificial neural networks. Due to the complex dynamical behaviors of chaotic systems, they have a wide range of applications such as secure communication [12]. Besides, compared with the integer-order chaotic system, the fractional-order chaotic system exhibits higher nonlinearity and larger key space due to the existence of fractional-order derivatives [13].

Research on synchronization is still attractive due to its potential applications in control processing and secure communication [14]. Various methods of chaotic synchronization have been proposed recently, such as impulse control [15], linear matrix inequalities (LMI) [16], sliding mode control [17] and many others based on observer theory to chaotic synchronization [18]. A well-known method is based on the state observer where the response system is designed to behave as an observer of the drive system [19]. Recently, many researchers have begun to pay attention to the synchronization problem of fractional-order chaotic systems, such as phase synchronization, antiphase synchronization, and generalized projective synchronization.

In the last few years, many papers on FHNN have been published [20,21,22,23,24,25,26,27]. Unfortunately, only a few of them studied the chaotic characteristics and generalized projective synchronization (GPS) schemes of time-delayed FHNN. Motivated by the discussions above, in this paper, we propose a new class of time-delayed FHNN and analyze the rich dynamics, then design its GPS scheme.

The remainder of this paper is organized as follows. In Section 2, the basic definition of fractional derivatives, the stability criterion of the fractional-order system, and the numerical algorithm of time-delayed FHNN are presented. In Section 3, the new time-delayed FHNN with three neurons is introduced. Its complex dynamics like chaos, periodicity, and stability are analyzed. Its chaotic nature is confirmed by phase portraits, bifurcation diagrams, and the Largest Lyapunov exponent. In Section 4 a GPS scheme between two identical time-delayed FHNN based on the state observer is introduced. Finally, in Section 5, some conclusions are given.

## 2. Preliminaries and Numerical Algorithm

### 2.1. Preliminaries

There exist some definitions of fractional derivatives; in this article, we will use the Caputo derivative due to its wider range of engineering applications. The Caputo derivation definition is as follows [1]:

(1) $$\begin{array}{c} D^{q}x\left(t\right)=D^{-(n-q)}\frac{d^{n}}{dt^{n}}x\left(t\right)=\frac{1}{\Gamma (n-q)}\int_{t0}^{t}{\left(t-\tau \right)}^{n-q-1}x^{n}\left(\tau \right)d\tau \end{array}$$

where q(q>0) is the fractional-order, $n-1<q<n\in Z^{+},$ and Γ(·) is the gamma function $\Gamma \left(x\right)=\int_{0}^{\infty }t^{x-1}e^{-t}dt.$

The stability theory of fractional derivative system is not the same as that of the integer system. For an n-dimensional fractional-order system:

(2) $$D^{q}\left(X\right)=g\left(X\right),$$

where $D^{q}\left(X\right)={\left(D^{q_{1}}\left(x_{1}\right),D^{q_{2}}\left(x_{2}\right),\cdots ,D^{q_{n}}\left(x_{n}\right)\right)}^{T},X=\left(x_{1},x_{2},\cdots ,x_{n}\right).$

Like the integer case, the roots of the equation g(x)=0 are the equilibrium points of this fractional system.

**Theorem** **1.** *[28] For an n-dimensional fractional-order system, if all the eigenvalues $\left(\lambda_{1},\lambda_{2},\cdots ,\lambda_{n}\right)$ of the Jacobian matrix of some equilibrium points satisfy:*

(3) $$\begin{array}{c} \left|\arg(\lambda_{i})\right|>a\pi /2,a=\max\left(q_{1},q_{2},\cdots ,q_{n}\right)\;\left(i=1,2,\cdots ,n\right). \end{array}$$

*then the system is asymptotically stable at one point.*

**Lemma** **1.** *If there exists one equilibrium point that is stable, then the fractional system will be stable; if and only if all equilibrium points are unstable, then the fractional system will be unstable, maybe periodic or chaotic.*

**Lemma** **2.** *For n = 3, the equilibrium point is called a saddle point with index two, if one of the eigenvalues $\lambda_{1}<0$ and the other two conjugate eigenvalues $\left|\arg(\lambda_{2})\right|=\left|\arg(\lambda_{3})\right|<q\pi /2$; the equilibrium point is called a saddle point with index one, if one of the eigenvalues $\lambda_{1}>0$ and the other two conjugate eigenvalues $\left|\arg(\lambda_{2})\right|=\left|\arg(\lambda_{3})\right|>q\pi /2$. Both of these two types of saddle points are unstable.*

### 2.2. Numerical Algorithm

Many numerical algorithms to solve the differential equations of fractional-order (FDE) have been proposed [29,30,31]. We will use the widely-used method, which is modified based on the Adams–Bashforth–Moulton predictor-corrector scheme, to solve time-delayed differential equations of fractional-order (FDDE) [31]. The method is described below.

Consider the general FDDE:

(4) $$\begin{array}{c} D_{t}^{q}y\left(t\right)=f\left(t,y\left(t\right),y\left(t-\tau \right)\right),t\in \left[0,T\right],q\in \left(0,1\right]\\ y(t)=m(t),t\in [-\tau ,0]. \end{array}$$

Consider a uniform grid $\left\{t_{n}=nh:n=-l,-l+1,\cdots ,-1,0,1,\cdots ,N\right\}$, in which l∈Z,N∈Z and l=τ/h,N=T/h. Let:

(5) $$y_{h}\left(t_{k}\right)=m\left(t_{k}\right),k=-l,-l+1,\cdots ,-1,0.$$

and denote:

(6) $$y_{h}\left(t_{k}-\tau \right)=y_{h}\left(kh-lh\right)=y_{h}\left(t_{k-l}\right),k=0,1,\cdots ,N.$$

Suppose that we have already obtained the approximations $y_{h}\left(t_{k}\right)\left(k=1,2,\cdots ,n\right),$ then we will calculate $y_{h}\left(t_{n+1}\right)$ according to:

(7) $$y\left(t_{n+1}\right)=m\left(0\right)+\frac{1}{\Gamma (q)}\int_{0}^{t_{n-1}}{\left(t_{n+1}-\zeta \right)}^{q+1}f\left(\zeta ,y\left(\zeta \right),y\left(\zeta -\tau \right)\right)d\zeta .$$

We obtain Equation (7) by applying fractional integration on both sides of (4). Then, we use approximations $y_{h}\left(t_{n}\right)$ for $y(t_{n})$. Thus, the corrector formula is as follows:

(8) $$\begin{array}{cc} y_{h}\left(t_{n+1}\right) & =m\left(0\right)+\frac{h^{q}}{\Gamma (q+2)}f\left(t_{n+1},y_{h}\left(t_{n+1}\right),y_{h}\left(t_{n+1}-\tau \right)\right)+\frac{h^{q}}{\Gamma (q+2)}\sum_{k=0}^{n}a_{k,n+1}f\left(t_{j},y_{h}\left(t_{j}\right),y_{h}\left(t_{j}-\tau \right)\right)\\ & =m\left(0\right)+\frac{h^{q}}{\Gamma (q+2)}f\left(t_{n+1},y_{h}\left(t_{n+1}\right),y_{h}\left(t_{n+1-l}\right)\right)+\frac{h^{q}}{\Gamma (q+2)}\sum_{k=0}^{n}a_{k,n+1}f\left(t_{j},y_{h}\left(t_{j}\right),y_{h}\left(t_{j-l}\right)\right), \end{array}$$

where:

(9) $$\begin{array}{c} a_{k,n+1}=\left\{\begin{array}{cc} n^{q+1}-\left(n-q\right){\left(n+1\right)}^{q}, & k=0\\ {\left(n-k-2\right)}^{q+1}+{\left(n-k\right)}^{q+1}\\ -2{\left(n-k+1\right)}^{q+1}, & 1<k<n\\ 1, & k=n+1 \end{array}\right. \end{array}$$

Then, we use a “predictor” $y_{h}^{P}\left(t_{n+1}\right)$ to replace the term $y_{h}\left(t_{n+1}\right)$ on the right side of (8).

where:

(10) $$\begin{array}{cc} y_{h}^{P}\left(t_{n+1}\right) & =m\left(0\right)+\frac{1}{\Gamma (q)}\sum_{k=0}^{n}b_{k,n+1}f\left(t_{k},y_{h}\left(t_{j}\right),y_{h}\left(t_{k}-\tau \right)\right)\\ & =m\left(0\right)+\frac{1}{\Gamma (q)}\sum_{k=0}^{n}b_{k,n+1}f\left(t_{k},y_{h}\left(t_{j}\right),y_{h}\left(t_{k-l}\right)\right),\\ b_{k,n+1} & =\frac{h^{q}}{q}\left({\left(n+1-k\right)}^{q}-{\left(n-j\right)}^{q}\right). \end{array}$$

In the remaining part of this paper, we use the algorithm above for numerical simulation and take the step size *h* = 0.01.

## 3. Dynamic Analysis of This New Time-Delayed FHNN

### 3.1. System Description

The time-delayed FHNN model can be presented as the following fractional-order differential equation:

(11) $$\begin{array}{cc} D_{t}^{q}\left(x_{i}\left(t\right)\right) & =-c_{i}x_{i}\left(t\right)+\sum_{j=1}^{n}W_{ij}f\left(x_{j}\left(t\right)\right)+\sum_{j=1}^{n}T_{ij}f\left(x_{j}\left(t-\tau_{ij}\right)\right)+I_{i},i=1,2,\cdots ,n,\\ x_{i}\left(t\right) & =m_{i}\left(t\right),t\in \left[-\tau ,0\right],0<q<1, \end{array}$$

where *q* is the Caputo differential operator, *n* represents the number of units in a Hopfield neural network, $c_{i}$ is a positive real number, $c_{i}$ represent the self-regulating coefficients of the neurons, $W_{ij}$ and $T_{ij}$ are real constant numbers, $W_{ij}$ represents the synaptic connection coefficient of the neuron *j* on the neuron *i* at time *t*, and $T_{ij}$ represents the synaptic connection coefficient of the neuron *j* on the neuron *i* at time $t-\tau_{ij}$. f(x) represents the activation to its incoming potentials of the unit *j* at time *t*, also known as the activation function, and $\tau_{ij}$ is the delay, while $I_{i}$ represent the external input.

In this article, we consider time-delayed FHNN with three neurons; then, System (11) can be rewritten with the vector form as follows:

(12) $$\begin{array}{cc} D_{t}^{q}\left(x\left(t\right)\right) & =-Cx(t)+Wf(x(t))+Tf(x(t-\tau ))+I\\ x(t) & =m(t),t\in [-\tau ,0],0<q<1, \end{array}$$

where $x\left(t\right)={\left(x_{1}\left(t\right),x_{2}\left(t\right),x_{3}\left(t\right)\right)}^{T}$, corresponds to the state vector at time *t* of three neurons, f(x(t))=tanh(x(t), f(x(t)), the activation function that we chose as the hyperbolic tangent, and f(x(t))=tanh(x(t),$m\left(t\right)={\left(0,0,0\right)}^{T}$, is the initial condition. $I={\left(0,0,0\right)}^{T}$ is the external input vector. The weight matrix largely determines the characteristics of the entire network, such as good memory characteristics, classification characteristics, and chaos characteristics.

After the elaborate design, our connection weight matrix is defined as follows:

(13) $$\begin{array}{c} C=\left(\begin{array}{ccc} 1 & 0 & 0\\ 0 & 1 & 0\\ 0 & 0 & 1 \end{array}\right)W=\left(\begin{array}{ccc} -1.5 & -19.9 & -3.2\\ 20 & -0.9 & 0.7\\ 1.4 & 2.79 & -2.3 \end{array}\right)T=\left(\begin{array}{ccc} 0 & 19.9 & 0\\ 0 & 0 & 0\\ 0 & 0 & 0 \end{array}\right) \end{array}$$

**Figure 1:**
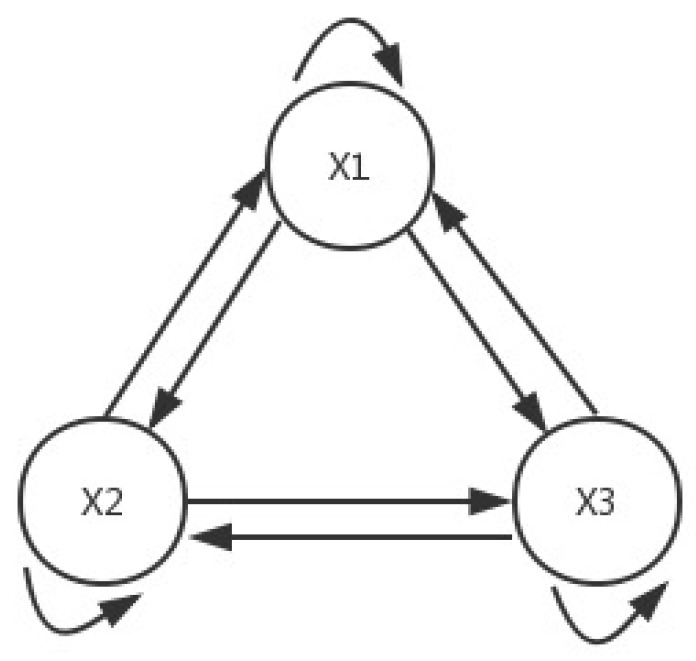
Connection topology of System (12).

The topology connection of System (12) is the full connection as Figure 1 shows.

### 3.2. Dynamic Analysis

Firstly, we consider the case in which the delay τ=0; then, System (12) becomes an FHNN with three neurons, and the system of equations becomes:

(14) $$\begin{array}{cc} \left(\begin{array}{c} D^{q}\left(x_{1}\left(t\right)\right)\\ D^{q}\left(x_{2}\left(t\right)\right)\\ D^{q}\left(x_{3}\left(t\right)\right) \end{array}\right) & =\left(\begin{array}{c} -x_{1}\left(t\right)\\ -x_{2}\left(t\right)\\ -x_{3}\left(t\right) \end{array}\right)+\left(\begin{array}{ccc} -1.5 & 0 & -3.2\\ 20 & -0.9 & 0.7\\ 1.4 & 2.79 & -2.3 \end{array}\right)*\left(\begin{array}{c} \tanh(x_{1}\left(t\right))\\ \tanh(x_{2}\left(t\right))\\ \tanh(x_{3}\left(t\right)) \end{array}\right)\\ & =\left(\begin{array}{c} -x_{1}\left(t\right)\\ -x_{2}\left(t\right)\\ -x_{3}\left(t\right) \end{array}\right)+W^{*}*\left(\begin{array}{c} \tanh(x_{1}\left(t\right))\\ \tanh(x_{2}\left(t\right))\\ \tanh(x_{3}\left(t\right)) \end{array}\right) \end{array}$$

**Figure 2:**
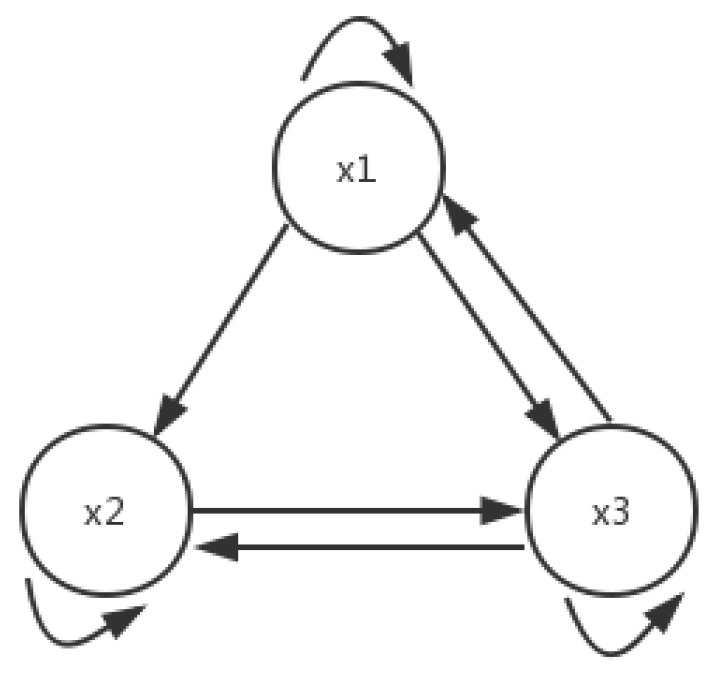
Connection topology of System (14).

Then, the topology connection of System (14) is no longer the full connection, which is shown in Figure 2.

**Figure 3:**
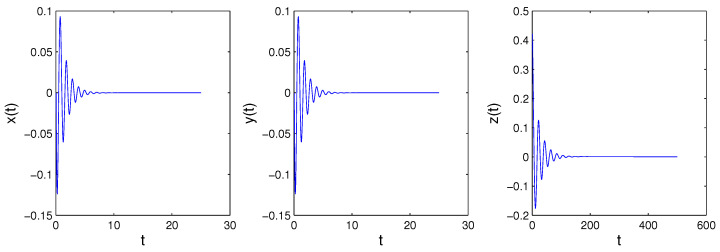
When *q* = 0.9, System (14) converges to the origin.

**Figure 4:**
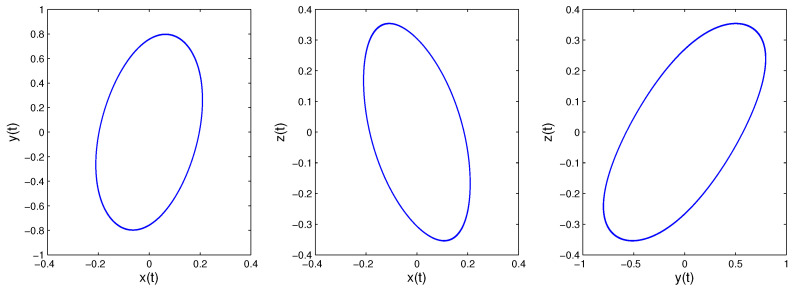
When *q* = 0.99; System (14)’s phase diagrams.

When we consider the equilibrium points of the fractional-order system, it is the same as the integer case. Because all principal minors of $-W^{*}$ are nonnegative, System (14) has a unique equilibrium point [32]. Obviously, the equilibrium point is the origin. The eigenvalues of the corresponding Jacobian matrix are $\lambda_{1}=-8.047,\lambda_{2,3}=0.174\pm 4.956i$. According to Lemma 1, if Condition (3) is satisfied, the system will be convergent. Otherwise, the system may be periodic or chaotic once Condition (3) is not satisfied. We can obtain $\arg\left(\lambda_{1}\right)=\pi ,\left|\arg(\lambda_{2})\right|=\left|\arg(\lambda_{3})\right|=1.536$. If $q>\frac{2}{\pi }\cdot \left|\arg(\lambda_{2})\right|=0.977$ is satisfied, the origin will be a saddle point with index two, the system may be periodic or chaotic. If $q<\frac{2}{\pi }\cdot \left|\arg(\lambda_{2})\right|=0.977$ is satisfied, the origin will be a stable point, and the system will be convergent. Figure 3 and Figure 4 are the simulation results of System (14) with order 0.9 and 0.99, respectively, consistent with the analysis results above.

**Figure 5:**
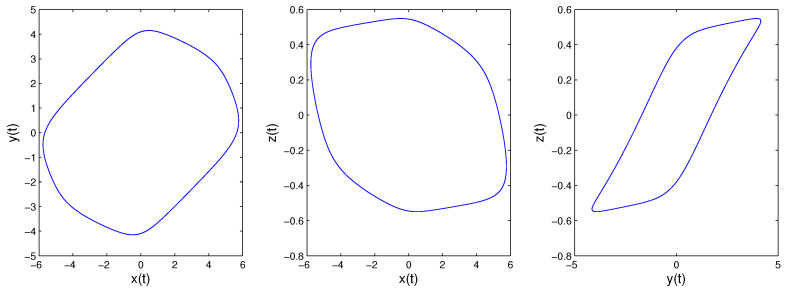
When *q* = 0.9, τ=0.5; System (13)’s phase diagrams.

**Figure 6:**
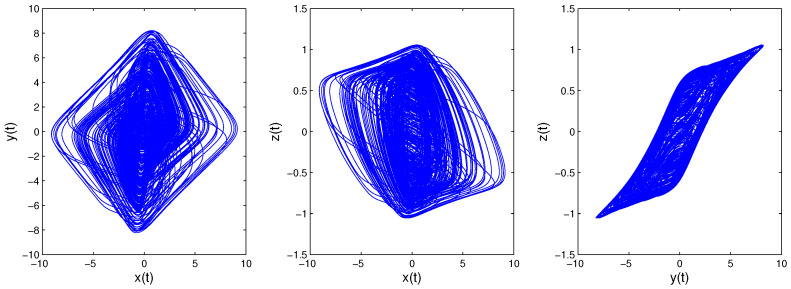
When *q* = 0.9, τ=1.8; System (13)’s phase diagrams.

**Figure 7:**
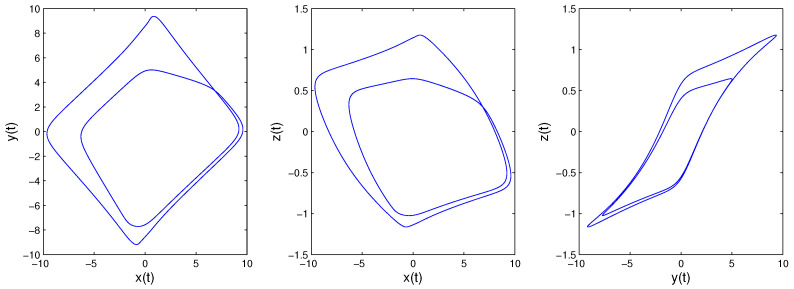
When *q* = 0.9, τ=3.3; System (13)’s phase diagrams.

**Figure 8:**
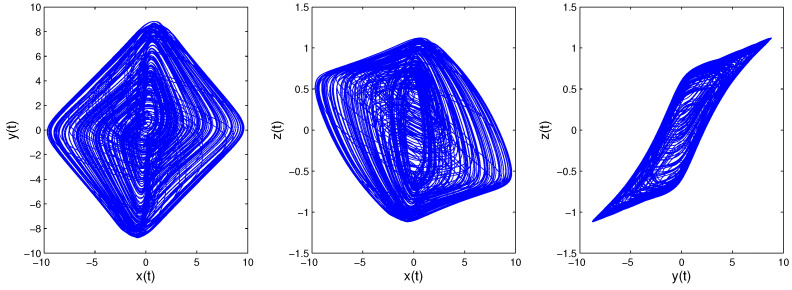
When *q* = 0.9, τ=4; System (13)’s phase diagrams.

**Figure 9:**
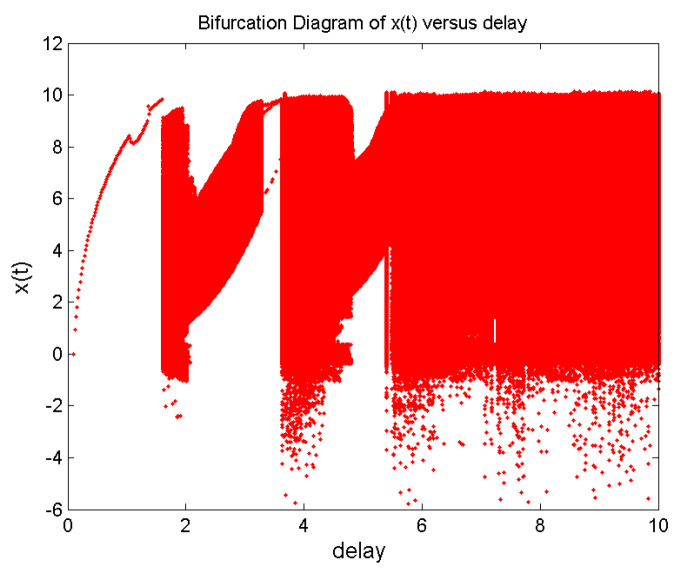
Bifurcation diagrams of x(t) versus delay τ.

**Figure 10:**
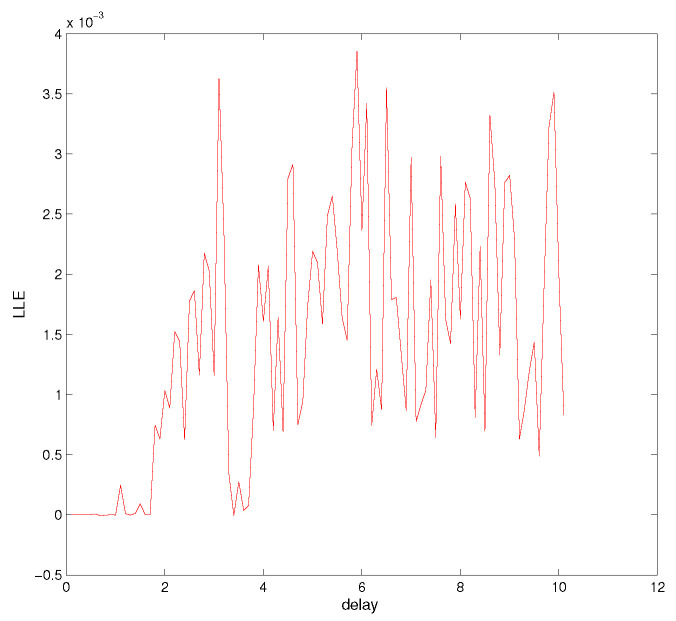
LLEversus delay τ.

Time-delay can cause changes in system stability [19]. Here, we consider system changes at different delays while fixing q=0.9. When τ=0.5, as shown in Figure 5, the system will be unstable, which is periodic; the system trajectory is periodic and oscillates around the equilibrium point. Increasing the delay to 1.8, we can learn from Figure 6 that the system becomes chaotic, one chaotic attractor exists in the system trajectory. If we continue increasing the delay, we can see the intermittent chaos phenomenon. When delay τ=3.3, the system trajectory, as shown in Figure 7, becomes periodic again. When increasing the delay to four, the system becomes chaotic again, as shown in Figure 8; the chaotic attractor exists once again. In order to see intermittent chaos intuitively, we draw the bifurcation diagrams of x(t) versus τ shown in Figure 9. In order to further confirm the phenomenon above, we calculate the largest Lyapunov exponents(LLE) of the above system by the TISEAN package [33], shown in Figure 10.

## 4. Generalized Projective Synchronization of Time-Delayed FHNN

In this section, we discuss the GPS of the system above. Synchronization of the fractional-order system based on the state observer has been reported in many articles [34,35,36,37]. We will extend this method to a class of time-delayed FHNN. A time-delayed FHNN system serves as the driving system, which can be rewritten as follows:

(15) $$\begin{array}{cc} D_{t}^{q}\left(x\left(t\right)\right) & =-Cx(t)+W\{f(x(t))+Zf(x(t-\tau ))\},\\ x(t) & =m(t),t\in [-\tau ,0],0<q<1,\\ u(t) & =Kx(t)+\{f(x(t))+Zf(x(t-\tau ))\}. \end{array}$$

where $x\left(t\right)={\left(x_{1}\left(t\right),x_{2}\left(t\right),\cdots ,x_{n}\left(t\right)\right)}^{T},f\left(x\left(t\right)\right)=\tanh\left(x\left(t\right)\right),m\left(t\right)={\left(0,0,\cdots ,0\right)}^{T},C,W$ and $Z\in R^{nxn}$ are constant matrices, u(t) is the synchronized vector signal, and $K\in R^{nxn}$ will be determined later.

The response system can be designed as follows:

(16) $$\begin{array}{cc} D_{t}^{q}\left(y\left(t\right)\right) & =-Cy(t)+W\{f(y(t))+Zf(y(t-\tau ))\}+W(u(t)-v(t)),\\ y(t) & =m(t),t\in [-\tau ,0],0<q<1,\\ v(t) & =Ky(t)+\{f(y(t))+Zf(y(t-\tau ))\}. \end{array}$$

where $y\left(t\right)={\left(y_{1}\left(t\right),y_{2}\left(t\right),\cdots ,y_{n}\left(t\right)\right)}^{T},f\left(y\left(t\right)\right)=\tanh\left(y\left(t\right)\right),m\left(t\right)={\left(0,0,\cdots ,0\right)}^{T},C,W$ and $Z\in R^{nxn}$ are constant matrices, v(t) is the observer predictor of u(t), and $K\in R^{nxn}$ will be determined later.

The GPS error signal of System (15) and System (16) can be written as e(t)=y(t)−εx(t);ε is the scaling factor, which is a non-zero real number. Obviously, if the scaling factor is selected as one, the synchronization will become complete synchronization. The fractional-order error signal is determined as follows:

$$D_{t}^{q}\left(e\left(t\right)\right)=D_{t}^{q}\left(y\left(t\right)-\varepsilon x\left(t\right)\right),$$

according to the basic properties of fractional-order operations, the error signal can be expanded as:

$$D_{t}^{q}\left(e\left(t\right)\right)=D_{t}^{q}\left(y\left(t\right)-\varepsilon x\left(t\right)\right)=D_{t}^{q}\left(y\left(t\right)\right)-\varepsilon D_{t}^{q}\left(x\left(t\right)\right),$$

then we replace the term $D_{t}^{q}\left(y\left(t\right)\right)$ and $D_{t}^{q}\left(x\left(t\right)\right)$ with System (16) and System (15), respectively. The error system becomes:

$$\begin{array}{cc} D_{t}^{q}\left(e\left(t\right)\right)= & -Cy(t)+W\{f(y(t))+Zf(y(t-\tau ))\}+W(\varepsilon u(t)-v(t))\\ & -\varepsilon \{-Cx(t)+W\{f(x(t))+Zf(x(t-\tau ))\}\}, \end{array}$$

now, we replace the term u(t) and v(t) with System (15) and System (16), respectively. The error system can be simplified as:

(17) $$\begin{array}{cc} D_{t}^{q}\left(e\left(t\right)\right) & =-Cy(t)+W\{f(y(t))+Zf(y(t-\tau ))\}+W\{\varepsilon \{Kx(t)+\{f(x(t))+Zf(x(t-\tau ))\}\}\\ & \;-Ky(t)-\{f(y(t))+Zf(y(t-\tau ))\}\}+\varepsilon Cx(t)-\varepsilon W\{f(x(t))+Zf(x(t-\tau ))\}\\ & =(-C-WK)(y(t)-\varepsilon x(t))\\ & =(-C-WK)e(t) \end{array}$$

System (15) and System (16) will achieve GPS if the error signal eventually stabilizes at the origin. We can easily get that the origin is one of the equilibrium points of System (17). According to Theorem 1 and Lemma 1, if all the eigenvalues $\left(\lambda_{1},\lambda_{2},\cdots ,\lambda_{n}\right)$ of the Jacobian matrix of origin satisfy:

(18) $$\left|\arg(\lambda_{i})\right|>q\pi /2,i=1,2,\cdots ,n,$$

then the error system will be asymptotically stable at one point. We can select some eigenvalues satisfying Inequality (18), because *C* and *W* are known system parameters, and applying some knowledge of matrix theory and with the aid of MATLAB, we can obtain the desired *K*.

For example, we use the system we proposed in Section 3.2 and set q=0.9. Values for the connection weight matrix *C*, *W*, and *Z* are as follow:

$$C=\left(\begin{array}{ccc} 1 & 0 & 0\\ 0 & 1 & 0\\ 0 & 0 & 1 \end{array}\right)W=\left(\begin{array}{ccc} -1.5 & -19.9 & -3.2\\ 20 & -0.9 & 0.7\\ 1.4 & 2.79 & -2.3 \end{array}\right)Z=\left(\begin{array}{ccc} 0 & -0.0021 & 0\\ 0 & -0.8365 & 0\\ 0 & -1.0159 & 0 \end{array}\right).$$

For the simplicity of calculation, we can select eigenvalues as: $\lambda_{1}=-1,\lambda_{2}=-2,\lambda_{3}=-3;$ at this time, Inequality (18) is satisfied. We can get the desired *K* by solving the following matrix equation:

$$-\left(\begin{array}{ccc} 1 & 0 & 0\\ 0 & 1 & 0\\ 0 & 0 & 1 \end{array}\right)-\left(\begin{array}{ccc} -1.5 & -19.9 & -3.2\\ 20 & -0.9 & 0.7\\ 1.4 & 2.79 & -2.3 \end{array}\right)*K=\left(\begin{array}{ccc} -1 & 0 & 0\\ 0 & -2 & 0\\ 0 & 0 & -3 \end{array}\right),$$

after some simple calculations, we obtain:

(19) $$K=\left(\begin{array}{ccc} 0 & 0.0489 & 0.0301\\ 0 & -0.0071 & 0.1126\\ 0 & 0.0212 & -0.7146 \end{array}\right).$$

Substituting Equation (19) into Equation (17), the error system becomes:

(20) $$\left(\begin{array}{c} D_{t}^{0.9}\left(e_{1}\left(t\right)\right)\\ D_{t}^{0.9}\left(e_{2}\left(t\right)\right)\\ D_{t}^{0.9}\left(e_{3}\left(t\right)\right) \end{array}\right)=\left(\begin{array}{ccc} -1 & 0 & 0\\ 0 & -2 & 0\\ 0 & 0 & -3 \end{array}\right)\left(\begin{array}{c} e_{1}\left(t\right)\\ e_{2}\left(t\right)\\ e_{3}\left(t\right) \end{array}\right).$$

**Figure 11:**
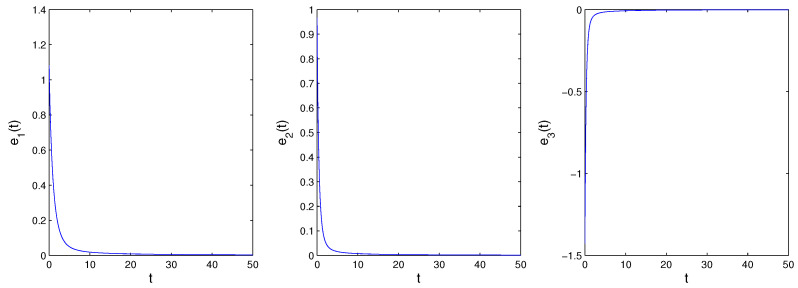
The time evolution of the error signal.

Obviously, System (20) will stabilize at the origin. Figure 11 is the time evolution of the error signal, which shows that System (20) will converge to the origin. That is to say, System (15) and System (16) achieve GPS.

## 5. Conclusions

In this paper, a novel time-delayed FHNN with three neurons is proposed. The rich dynamical behaviors of this new system are analyzed theoretically and verified by numerical simulations. Without delay, this new system presents stability or periodicity with different fractional-orders. Along with the increase of delay, the system presents an intermittent chaos phenomenon when the fractional-order is fixed. The GPS of two time-delayed FHNN with the same structure are achieved by using a synchronization method based on the state observer. The novel time-delayed FHNN can be used as a new chaos generator, which can be applied in many engineering applications such as secure communication. How to implement this system and synchronization method with actual circuits and how to apply them in secure communication will be discussed in the future.

## References

[1] Podlubny I. (1999). Fractional Differential Equations.

[2] Arena P., Caponetto R., Fortuna L., Porto D. (2000). Nonlinear Noninteger Order Circuits and Systems—An Introduction.

[3] Cafagna D. (2007). Fractional Calculus: A Mathematical Tool from the Past for Present Engineers. IEEE Ind. Electron. Mag..

[4] Magin R.L. (2004). Fractional calculus in bioengineering. Crit. Rev. Biomed. Eng..

[5] Manabe S. (2001). A Suggestion of Fractional-Order Controller for Flexible Spacecraft Attitude Control. Nonlinear Dyn..

[6] Kempfle S., Schäfer S., Beyer H. (2002). Fractional Calculus via Functional Calculus: Theory and Applications. Nonlinear Dyn..

[7] Hopfield J.J. (1984). Neurons with graded response have collective computational properties like those of two-state neurons. Proc. Natl. Acad. Sci. USA.

[8] Li H.L., Cao J.D., Jiang H.J., Alsaedi A. (2018). Finite-time synchronization of fractional-order complex networks via hybrid feedback control. Neurocomputing.

[9] Guevara M.R., Glass L., Mackey M.C., Shrier A. (1983). Chaos in neurobiology. IEEE Trans. Syst. Man Cybern..

[10] Babloyantz A., Lourenco C. (1996). Brain chaos and computation. Int. J. Neural Syst..

[11] Freeman W.J. (1991). The physiology of perception. Sci. Am..

[12] Mata-Machuca J.L., Aguilar-Lopez R. (2018). Adaptative synchronization in multi-output fractional-order complex dynamical networks and secure communications. Eur. Phys. J. Plus.

[13] Kiani B.A., Fallahi K., Pariz N., Leung H.A. (2009). Chaotic secure communication scheme using fractional chaotic systems based on an extended fractional Kalman filter. Commun. Nonlinear Sci. Numer. Simul..

[14] Tlelo-Cuautle E., Gerardo D.L.F.L., Pham V.T., Volos C., Jafari S., Quintas-Valles A.D.J. (2017). Dynamics, FPGA realization and application of a chaotic system with an infinite number of equilibrium points. Nonlinear Dyn..

[15] Yang C.H., Ge Z.M., Chang C.M., Li S.Y. (2010). Chaos synchronization and chaos control of quantum-CNN chaotic system by variable structure control and impulse control. Nonlinear Anal. Real World Appl..

[16] Chen L.P., Chai Y., Wu R.C. (2011). Linear matrix inequality criteria for robust synchronization of uncertain fractional-order chaotic systems. Chaos.

[17] Singh J.P., Roy B.K. (2018). Second order adaptive time varying sliding mode control for synchronization of hidden chaotic orbits in a new uncertain 4-D conservative chaotic system. Trans. Inst. Meas. Control.

[18] Carbajal-Gomez V.H., Tlelo-Cuautle E., Sanchez-Lopez C., Fernandez-Fernandez F.V. (2018). PVT-Robust CMOS Programmable Chaotic Oscillator: Synchronization of Two 7-Scroll Attractors. Electronics.

[19] Grassi G., Mascolo S. (1997). Nonlinear observer design to synchronize hyperchaotic systems via a scalar signal. IEEE Trans. Circ. Syst. I.

[20] Wang H., Yu Y.G., Wen G.G., Zhang S., Yu J.Z. (2015). Global stability analysis of fractional-order Hopfield neural networks with time delay. Neurocomputing.

[21] Huang X., Wang Z., Li X.L. (2013). Nonlinear Dynamics and Chaos in Fractional-Order Hopfield Neural Networks with Delay. Adv. Math. Phys..

[22] Pu Y.F., Yi Z., Zhou J.L. (2017). Fractional Hopfield Neural Networks: Fractional Dynamic Associative Recurrent Neural Networks. IEEE Trans. Neural Netw. Learn. Syst..

[23] Xi Y.G., Yu Y.G., Zhang S., Hai X.D. (2018). Finite-time robust control of uncertain fractional-order Hopfield neural networks via sliding mode control. Chin. Phys. B.

[24] Zhang S., Yu Y.G., Geng L.L. (2017). Stability Analysis of Fractional-Order Hopfield Neural Networks with Time-Varying External Inputs. Neural Process Lett..

[25] Huang C.D., Cao J.D., Ma Z.J. (2016). Delay-induced bifurcation in a tri-neuron fractional neural network. Int. J. Syst. Sci..

[26] Chen L.P., Qu J.F., Chai Y., Wu R.C., Qi G.Y. (2013). Synchronization of a Class of Fractional-Order Chaotic Neural Networks. Entropy.

[27] Kaslik E., Sivasundaram S. (2012). Nonlinear dynamics and chaos in fractional-order neural networks. Neural Netw..

[28] Munoz-Pacheco J.M., Zambrano-Serrano E., Volos C., Jafari S., Kengne J., Rajagopal K. (2018). A New Fractional-Order Chaotic System with Different Families of Hidden and Self-Excited Attractors. Entropy.

[29] Zúñiga-Aguilar C.J., Coronel-Escamilla A., Gómez-Aguilar J.F., Alvarado-Martínez V.M., Romero-Ugalde H.M. (2018). New numerical approximation for solving fractional delay differential equations of variable order using artificial neural networks. Eur. Phys. J. Plus.

[30] Bhalekar S., Daftardar-Gejji V. (2011). A predictor corrector scheme for solving nonlinear delay differential equations of fractional-order. J. Fract. Calculus Appl..

[31] Zúñiga-Aguilar C.J., Romero-Ugalde H.M., Gomez-Aguilar J.F., Escobar-Jimenez R.F., Valtierra-Rodriguez M. (2017). Solving fractional differential equations of variable-order involving operators with Mittag-Leffler kernel using artificial neural networks. Chaos Solitons Fract..

[32] Forti M., Marini M., Manetti S. (1994). Necessary and sufficient condition for absolute stability of neural networks. IEEE Trans. Circuits Syst. I Fund. Theory Appl..

[33] Hegger G., Kantz H., Schreiber T. (1999). Practical implementation of nonlinear time series methods: The TISEAN package. Chaos.

[34] Ghosh D. (2010). Nonlinear active observer-based generalized synchronization in time-delayed systems. Nonlinear Dyn..

[35] Cafagna D., Grassi G. (2015). Fractional-order systems without equilibria: The first example of hyperchaos and its application to synchronization. Chin. Phys. B.

[36] Jia Y.Q., Jiang G.P. (2017). Chaotic system synchronization of state-observer-based fractional-order time-delay. Acta Phys. Sin..

[37] Liu L., Liang D.L., Liu C.X. (2012). Nonlinear state-observer control for projective synchronization of a fractional-order hyperchaotic system. Nonlinear Dyn..

